# Report on the International Symposium on Hepatitis E, Seoul, South Korea, 2010

**DOI:** 10.3201/eid1805.111916

**Published:** 2012-05

**Authors:** Thomas F. Wierzba, Ursula Panzner

**Affiliations:** International Vaccine Institute, Seoul, South Korea

**Keywords:** Hepatitis, viruses, water-borne hepatitis, rHEV, HEV 239, HEV genomes, sanitation, poverty, South Korea, symposium

Hepatitis E virus (HEV), transmitted through the fecal–oral route, was first identified during waterborne epidemics of acute hepatitis in Kashmir and New Delhi, India, in 1980 (*1**–**3*). The virus was later characterized as a spherical virion of 27–34 nm in diameter with a nonenveloped, positive-sense, single-stranded RNA genome of ≈7.2 kb (*4*) and classified into 4 genotypes (*1**,**2**,**5**,**6*) that appear to have specific global distributions (*4*). Genotypes 1 and 2 are primarily responsible for HEV infections in humans; genotypes 3 and 4 can be found in animals and humans.

Clinical manifestations of HEV infection range from asymptomatic to fulminant hepatic failure and death. Symptomatic episodes are common in adolescents and adults; pregnant women have the highest attack rates (*1*). Although HEV infection results in low case-fatality rates (0.5%–4.0%) in the general population, HEV causes death in 10%–42% of infected pregnant women (*5**,**6*).

HEV epidemics occur primarily in areas with poor hygiene and poor sanitation, including countries in Asia, the Middle East, Africa, and Latin America. Despite the growing evidence of global effects of HEV, awareness of the public health implications of HEV infection remains low because of questions regarding its epidemiology; its health, economic, and social costs; and its virology and pathogenesis. The disease and its case-fatality rate probably are underestimated because HEV infections are not clinically distinguishable from those caused by other hepatitis viruses. Validated, commercially available diagnostic assays are needed, and existing assays vary substantially for sensitivity (72%–98%) and specificity (79%– 96%).

During September 15–16, 2010, the International Vaccine Institute and the World Health Organization (WHO), with sponsorship from the US Centers for Disease Control and Prevention and the Bill & Melinda Gates Foundation, hosted the International Symposium on Hepatitis E in Seoul, South Korea. International experts (Table), including virologists, immunologists, molecular biologists, epidemiologists, clinicians, and policy makers, gathered to review published and unpublished epidemiologic, clinical, and laboratory data and to discuss control strategies. Participants considered, in particular, the impact of HEV, the 2 hepatitis E vaccine candidates, and outlined activities to characterize hepatitis E epidemiology and support vaccine development and other prevention strategies.

**Table:** Investigators who presented at the International Symposium on Hepatitis E, Seoul, South Korea, September 15–16, 2010

Investigator	Institute/organization
Dr Rakesh Aggarwal	Sanjay Gandhi Postgraduate Institute of Medical Sciences
Dr Alexander Andjaparidze	Former WHO Representative in Nepal
Dr R. Holland Cheng	University of California Davis
Dr Michael O. Favorov	Former International Vaccine Institute
Dr Saeed Hamid	Aga Khan University
Dr Scott D. Holmberg	US Centers for Disease Control and Prevention
Dr Yvan J-F Hutin	Office of the WHO Representative in China
Dr Makumba Issa	Ministry of Health of Uganda
Dr Nassim Kamar	Toulouse University Hospital
Dr Yury E. Khudyakov	US Centers for Disease Control and Prevention
Dr Mohammad S. Khuroo	Digestive Diseases Centre
Dr Alain B. Labrique	Johns Hopkins Bloomberg School of Public Health
Dr Stephen P. Luby	International Centre for Diarrhoeal Disease Research Bangladesh
Dr Helene Norder	Swedish Institute for Infectious Disease Control
Ms Ursula Panzner	International Vaccine Institute
Dr Robert H. Purcell	National Institute of Allergy and Infectious Diseases, National Institutes of Health
Dr David B. Rein	Public Health Economics Program
Dr Robert McNair Scott	formerly at Walter Reed AFRIMS Research Unit Nepal (WARUN)
Dr James Wai-Kuo Shih	Xiamen University
Dr Chong-Gee Teo	US Centers for Disease Control and Prevention
Dr Eyasu H. Teshale	US Centers for Disease Control and Prevention
Dr Theodore F. Tsai	Novartis Vaccines
Dr Nazira Usmanova	US Centers for Disease Control and Prevention, Central Asia Regional Office
Dr John W. Ward	US Centers for Disease Control and Prevention
Dr Ole Wichmann	Robert Koch Institute
Dr Steven Wiersma	World Health Organization
Dr Thomas F. Wierzba	International Vaccine Institute

The symposium presenters shared data that showed that HEV infection is endemic to industrialized and developing countries. WHO shared results of its 2010 Global Burden of Diseases, Injuries and Risk Factors study, which found that 63 countries had reported outbreaks or sporadic cases and 73 countries reported seroprevalence >10%.

During the session on the significance of hepatitis E as a public health threat in developing and other countries, HEV genotypes 1 and 2 were reported to be associated with large epidemics and high case-fatality rates, and to have induced ≈15 million symptomatic and 13 million asymptomatic cases in 2005. Other data presented indicated that the impact of HEV is high in many of the world’s largest population settings with poor hygiene and sanitation, particularly countries in Africa, Latin America, and southern and central Asia. Sporadic cases were reported in industrialized countries (e.g., Germany, Sweden, and the United States), and serologic studies indicated that prevalence in these populations was higher than previously thought.

In industrialized countries, autochthonous cases are caused by genotypes 3 and 4. These genotypes are associated with asymptomatic and chronic disease and are probably associated with zoonotic transmission through ingestion of HEV-infected meat. Illness caused by genotype 1 in these countries was imported.

After establishing the epidemiology of HEV, the symposium focused on the loss of life it causes. Pregnant women, immunosuppressed patients, and persons who have chronic liver disease constitute high-risk groups. Presenters discussed studies conducted in Bangladesh during 2001–2006 and Uganda during 2007 that reported pregnant women had the highest rate of death from HEV. In the Uganda outbreak, the case-fatality rate for pregnant women was 8.3% (1.6% of overall deaths). Suggested risk factors were poor sanitation, hygiene, and living conditions. Vertical transmission (mother to child) was also suggested, although further investigation is needed. Chronic liver disease has been associated with high rates of illness and death from HEV superinfection, and chronic HEV-associated hepatitis can develop in immunosuppressed patients. Data based on disease modeling presented at the symposium suggest that HEV is associated with 271,000 deaths annually, which is 10-fold more than the annual number of deaths from hepatitis A virus. Because many who die are young adults, many life-years are lost to HEV illness. Further estimates on the basis of modeling predict an annual global impact caused by genotypes 1 and 2 of 21.6 million symptomatic cases and 12,203 stillbirths. The actual numbers might be larger if genotypes 3 and 4 also contribute meaningfully.

As main preventive tools, 2 vaccines were discussed: the recombinant HEV vaccine developed by GlaxoSmithKline (Raleigh-Durham, NC, USA), and the recombinant HEV 239 vaccine developed by Xiamen Innovax Biotech (Xiamen, People’s Republic of China). Although both vaccines were shown to be safe and efficacious (protective efficacy >90%), further studies are needed to demonstrate safety and long-term efficacy in high-risk persons and populations living in HEV-endemic areas. Besides further clinical development of these vaccines, public health demand, feasibility, and cost-effectiveness must be demonstrated and financing made available in developing countries. Furthermore, these tasks must be accomplished in light of limited interest and resources for HEV vaccine development in industrialized countries.

In conclusion, participants made an international call to action against HEV infection and agreed that advocacy is needed to increase awareness of hepatitis E as a disease that can adversely affect public health, and to position hepatitis E as a neglected disease of the poor, which is potentially preventable by vaccines. Priorities are to improve quality and availability of HEV diagnostics and develop standardized surveillance methods in the absence of a diagnostic standard. Participants also prioritized research to demonstrate the applicability of vaccine candidates and data collection required for their WHO prequalification. Looking ahead, when hepatitis E vaccines become available, vaccine demonstration projects should be implemented in well-characterized populations and supported by reliable diagnostic tools.

Symposium participants recommended the formation of an international hepatitis E collaboration to provide a multicountry, multidisciplinary platform for initiatives in research, policy, and advocacy. This collaboration would initially focus on supporting the development and evaluation of HEV diagnostics; developing standardized population- and hospital-based disease surveillance to conduct epidemiologic studies of high-risk populations; developing partnerships with industry to facilitate clinical trials of candidate hepatitis E vaccines and study cost-effectiveness of different vaccine strategies; and increasing global awareness, especially of opinion leaders and decision makers, on the threat of hepatitis E infection.
